# Reconnecting with Joseph and Augusta Dejerine: 100 years on

**DOI:** 10.1093/brain/awx225

**Published:** 2017-09-15

**Authors:** Claude J Bajada, Briony Banks, Matthew A Lambon Ralph, Lauren L Cloutman

**Affiliations:** Neuroscience and Aphasia Research Unit (NARU), Division of Neuroscience and Experimental Psychology, School of Biological Sciences, The University of Manchester, UK

## Abstract

Bajada *et al.* mark the centenary of Joseph Dejerine’s death by demonstrating the continuing relevance of his research with his long-standing collaborator, Augusta Dejerine-Klumpke, on the white matter pathways of the brain to modern-day connectional anatomy. A first English translation of the original work is provided in the Supplementary Materials.

## Introduction

Joseph Dejerine passed away on 28 February 1917 in the midst of a world at war. One hundred years later we celebrate the legacy of this pioneer in neuroscience. In 1895, Joseph Jules Dejerine published the first volume of the seminal work, *Anatomie des centres nerveux*; volume 2 was published in 1901. In a major section of this tome (vol. 1 pp. 749–80), Joseph Dejerine and his wife and long-term collaborator, Augusta Dejerine-Klumpke, produced a treatise on the white matter pathways of the brain, composed of anatomical descriptions of meticulous detail and beautiful illustration (drawn by H. Gillet) that reflected a combination of the most advanced methodologies of the day and a review of leading neuroscientific research. We have selected and focused this specific output (which is provided for the first time as an English translation in the Supplementary material) from the many that the Dejerines published because its ideas and findings continue to be of relevance to modern neuroscience researchers today; especially those with an interest in connectional anatomy.

## The Dejerines: a short history

Born in Geneva, Joseph Jules Dejerine moved to Paris in 1871 in order to start his medical career. He was a founding member of the French Neurological Society and proceeded to become the chair of neurology at ‘La Salpêtrière’, a position previously held by Jean Martin Charcot. In his 67 years, Dejerine made great contributions to the medical and scientific community, of which *Anatomie des centres nerveux* was but one. He became one of the most eminent neurologists of his era. He was a pioneer in the study of neurological conditions and the structural and functional anatomy the brain. His resulting legacy includes many syndromes eponymously named after him; arguably the most famous of which is the medial medullary syndrome (often referred to as ‘Dejerine syndrome’). Dejerine passed away in 1917 after suffering from Bright’s disease (nephritis) (Schurch and Dollfus, 1998).

**Figure 1:**
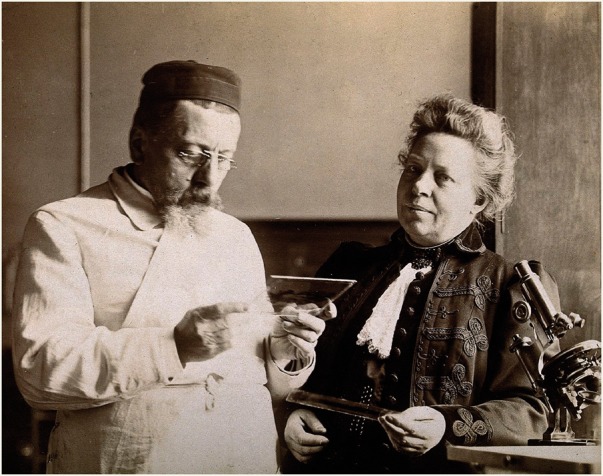
**Joseph Jules Dejerine and his wife Augusta Dejerine-Klumpke.** ©Wellcome Library London.

As with many great deeds, such as those told in the tales of Greek mythology, Joseph Dejerine’s success could not have been accomplished without the aid of a great woman. Alas, like Ariadne’s role in helping Theseus to slay the Minotaur, the role of Dejerine’s wife and long-term collaborator, Augusta Dejerine-Klumpke, has also often been overshadowed by that of her husband. Augusta Dejerine-Klumpke was a great neurologist in her own right who, in her husband’s words, ‘collaborated assiduously’ with him in the preparation of their masterpiece *Anatomie des centres nerveux* (Fig. 1). Augusta Marie Dejerine-Klumpke was born in San Francisco in 1859. She moved to Switzerland when she was 11 and eventually to Paris. Here she pursued her medical studies and became the first female ‘Interne des Hopitaux’ in France. Like her husband, Dejerine-Klumpke made a lasting impression within the field of medicine and was bestowed several honours for her work. Indeed, every medical student will be familiar with her for a syndrome that arises from damage to the inferior roots of the brachial plexus; the famous Klumpke’s palsy. She passed away 10 years after her husband, in 1927 (Schurch and Dollfus, 1998).

The Dejerines lived in a golden age for neurology, studying and working alongside, but not always in harmony with, some of the great masters of the time including Charcot, Pierre Marie and Vulpian. They published many works in their career but none as grand as their master work, the two-volume *Anatomie des centres nerveux*.

## A golden age for white matter neuroanatomy

The 19th century was a time of prolific work in connectional neuroanatomy. It was the era during which most of the white matter fasciculi known today were first dissected and described. It was also the time during which Paul Broca suggested that higher cognitive functions could be localized within the cortex, and Carl Wernicke proposed the first network approach to functional neuroanatomy. This connectional approach posited that (i) regions associated with specific cognitive functions dynamically interacted with one another to produce a more complex function; and (ii) resultantly, higher cognitive impairment resulted not only from damage to cortical regions but also to the connections between them. This concept was expanded upon by Dejerine in his view on pure alexia, which he conceptualized as a disconnection syndrome (Dejerine, 1892). It is worth noting, however, that Wernicke disagreed with the Dejerines’ view since it required the higher function of reading to be cortically localized to a ‘visual verbal centre’ within the angular gyrus (Catani and ffytche, 2005).

With the relatively recent emergence of diffusion MRI and related tractography techniques, modern neuroscience has been reconnecting with the work of the 19th century masters. A search of current literature will reveal a rapidly increasing interest in the association of specific cognitive functions to the white matter tracts of the brain. Despite the plethora of functional association studies, there remain many controversies surrounding the origin, course, termination and even sometimes the very existence of these tracts (Bajada *et al.*, 2015). Understanding the structure of the white matter that forms the brain’s circuitry remains an important goal for researchers who are interested in elucidating the brain’s function, as well as clinicians who would like to predict a patient’s outcome when a lesion occurs in one of these fasciculi or to avoid long-term deficits after neurosurgery (Duffau, 2015).

With this in mind, and at such a poignant time as the 100th anniversary of the passing of Joseph Dejerine, we highlight the legacy of two pioneers in the subject and revive their descriptions of the long association fasciculi of the cerebral cortex. These descriptions were based on an in-depth study of the white matter of the human brain, based on a convergence of methodologies available at the time including gross dissection, histological preparations, animal experimentation and a thorough contemporary review of known findings. A full translation (from its original French) of the section regarding the Long Association Fasciculi from *Anatomie des centres nerveux* is made available in the online Supplementary material that accompanies this article. For the remainder of this article we focus upon some of the key themes and findings, highlighting both striking parallels between the original work and modern descriptions, as well as 19th century notions that may have important implications for contemporary basic and clinical neuroscience.

## The long association fasciculi: past and present

The Dejerines described five long association fasciculi, all of which are still discussed in the modern literature, namely the cingulum, uncinate fasciculus, superior longitudinal/arcuate fasciculus, occipito-frontal fasciculus, and the inferior longitudinal fasciculus. While we leave the reader to examine the full translation for an in-depth description of the tracts, we explore three key discussion points.

- The first section explores the concept of a multi-component tract; the conglomeration of fibres that underlie different functional networks.
- The second section focuses on regions of white matter tracts outlined by the Dejerines but subsequently lost to the literature, either in the 19th or 20th century, and some current conceptualizations of these.
- The third section highlights the Dejerines’ contributions to long-standing debates, some of which are still unresolved in the contemporary literature.

Looking with a modern eye (and with modern methods) at the historical anatomical tract descriptions provided by the Dejerines, it is clear that some are incomplete and others inaccurate. However, such assertions could be argued to ring true for some modern anatomical studies of the brain’s major white matter tracts, and much of Dejerine’s work still holds true today. In addition, their descriptions offer an insight into the thoughts of two 19th century experts on topics that are still discussed and debated today.

### What (little) tracts are made of

Tracts can be conceptualized in one of two different ways, each of which can be likened to the wiring within a house. The first conceptualization posits that a tract is composed primarily of fibres that emerge from the same subregion, continue together along a single path and then terminate in the same destination. This is very much like the power cord of an electrical appliance through which positive, negative and earth wires travel together, therein providing the conduit from the power supply to the appliance. The second conceptualization, and the one promoted by the Dejerines in their anatomical descriptions (although they were not the first to do so), views a tract as a collection of fibres, from different sources and potentially carrying different information, which have been amalgamated together within a confined, shared neuroanatomical envelope (i.e. the tract). Under this view, a neural fibre may enter and exit a tract at any point along its trajectory. Accordingly, not only is a tract composed of long fibres that directly connect beginning and end points (as per the first conceptualization), but also of shorter fibres that connect the different regions that the tract passes along its trajectory. This second conceptualization is akin to a duct or trunk within a house in which wiring from the kitchen, bathroom, living room, etc. may be bundled together within a small space so they can be made to pass efficiently throughout the house. If one accepts this conception, then single tracts may be involved in very different functional networks. Hence, uncovering tract subcomponents, in addition to the major tract body, is an important step in understanding the functional associations of each tract. Furthermore, from a clinical neuroscience perspective, this second conceptualization would imply that the exact sequelae of tract damage may vary according to its position along each tract.

Examples of conceiving the tract as a shared neural wiring conduit occur frequently in the Dejerines’ descriptions. For example, they describe the cingulum as an arched fasciculus on the medial aspect of the brain, yet note that it is composed of smaller bundles of fibres:

‘Dissections show that the cingulum is not formed of fibres that extend the full length of the fasciculus, but of relatively short fibres. These short fibres are curved at both ends to penetrate white matter of the surrounding gyri and, for part of their trajectory, constitute the cingulum of Burdach.’

Likewise, the Dejerines’ conceptualization of the occipitofrontal fasciculus, the arcuate fasciculus and the inferior longitudinal fasciculus reinforces the idea of a tract as a multi-component shared conduit. For example, they describe the occipitofrontal fasciculus as one that ‘like all long association fasciculi, it is formed of fibres of unequal length that only belong to the occipito-frontal fasciculus for part of their trajectory’. The Dejerines described the arcuate fasciculus as a tract that is almost entirely composed of short fibres that connect nearby gyri, none of which traverse the entire course of the tract. Once again, although current evidence points to long range fibres being present within the arcuate fasciculus, the concept of multiple components remains.

Finally, in their description of the inferior longitudinal fasciculus, the Dejerines inculcate the concept that tracts are made up of fibres of differing lengths including long-range connections:

‘Like all long association fasciculi, the inferior longitudinal fasciculus comprises a complex system of fibres of unequal lengths. However, secondary degeneration, resulting from localised cortical lesions of the occipital lobe, has shown us that this fasciculus contains a large number of long fibres whose degeneration can be seen in the white matter of the temporal lobe.’

Although the exact descriptions of many of the association fasciculi have been challenged by later evidence (and indeed, continue to be revised and refined), the concept of a tract comprising fibres of multiple lengths remains firm.

### Lost and found

In the 19th century, anatomists primarily performed dissections and microscopic sections on post-mortem human brains. In contrast, much late 20th century neuroanatomical work used axonal tracer studies in non-human primates, or more advanced neuroimaging techniques. This difference in both methodology and/or species has contributed to discrepancies and disagreements in the literature regarding the composition of some of the main association tracts. This has led to key fibre bundle components delineated by the Dejerines’ historical work being lost and rediscovered by modern neuroscience, as well as components overlooked by the Dejerines being found.

**Figure 2:**
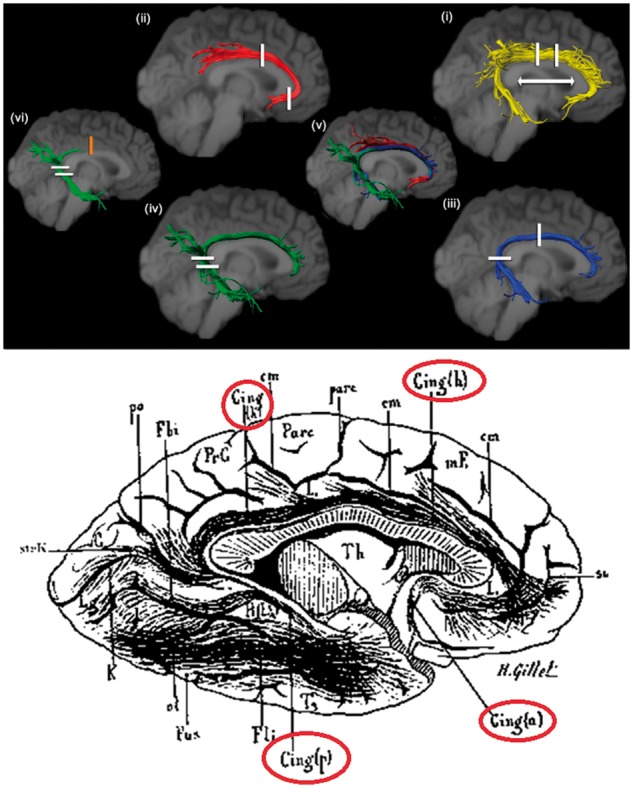
**The cingulum.** *Top*: Reconstruction using MRI diffusion tractography of the entire cingulum (yellow), and its subdivisions as identified by Jones *et al.* (2013). *Bottom*: Illustration by Gillet from *Anatomie des centres nerveux* depicting several subcomponents of the cingulum as described by the Dejerines; coloured circles highlight the different subcomponents of the cingulum roughly corresponding to the coloured components in Jones *et al.* (2013).

Using modern *in vivo *diffusion MRI tractography techniques, contemporary neuroscientists have re-examined many of the tracts originally discussed in both the 19th century dissection and the 20th century tract tracing literature, rediscovering the multi-element nature of key association tracts. For example, the Dejerines conceptualized the cingulum as being composed of a series of comparatively short-range tract subcomponents. Jones *et al.* (2013) reconstructed the cingulum and demonstrated that is composed of fibres of different lengths and several subcomponents, such as the subgenual, retrosplenial and parahippocampal subdivisions. Figure 2 depicts a comparison of the cingulum as described by the Dejerines and the modern delineation of the tract. However, while the multi-component nature of the cingulum has been rediscovered by modern neuroscience, it is also true to say that the long-range fibres, which also comprise the cingulum, were originally ‘lost’ by the Dejerines and have now been ‘found’.

**Figure 3:**
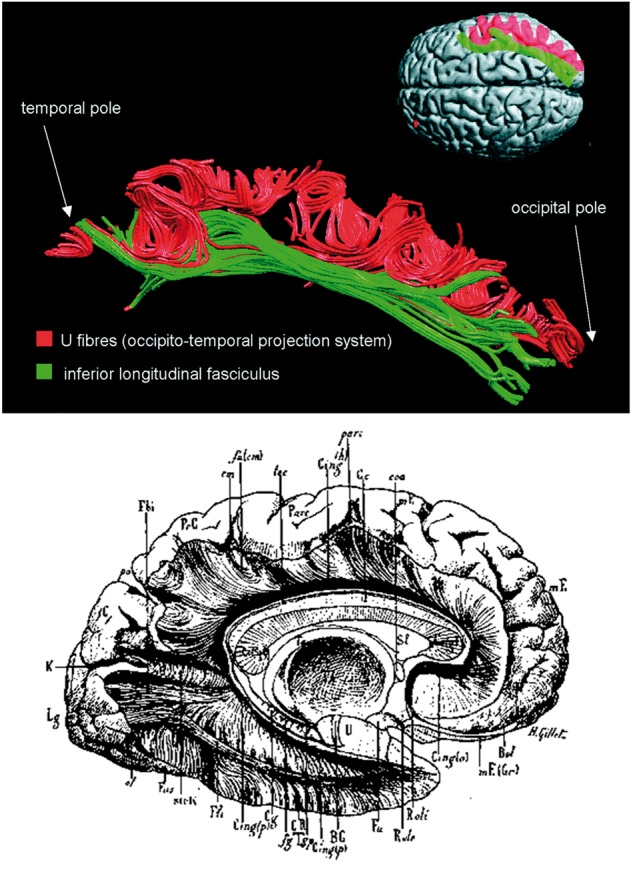
**The inferior longitudinal fasciculus and the occipito-temporal projection system.** *Top*: A modern reconstruction of the long-range fibres within the inferior longitudinal fasciculus alongside its U-fibre stream as delineated by Catani *et al.* (2003). Reproduced with permission of Oxford University Press. *Bottom*: A depiction of the inferior longitudinal fasciculus as described by the Dejerines in *Anatomie des centres nerveux*.

The inferior longitudinal fasciculus provides another example of modern tractography revisiting questions from the 19th and 20th century. The tract has a complex history and has, at various times, been conceptualized as either a single long tract or an occipitotemporal stream of short fibres connecting nearby gyri. During the 19th century, the great anatomists such as Charcot and Meynert believed that the inferior longitudinal fasciculus only had a projection fibre component (i.e. was not implicated in the cortical inter-regional connectivity underpinned by the association tracts). More recently, Tusa and Ungerleider (1985) posited that the pathway connecting the occipital and temporal cortices consisted only of a series of U fibres, ‘losing’ (or rather positing the elimination of) the tract altogether in favour of an occipitotemporal projection system. This is in stark contrast to the Dejerines’ description of the inferior longitudinal fasciculus as a trough-like tract that hugs the lateral ventricle connecting the occipital lobe to the temporal pole (see Supplementary material for an in-depth description). Indeed, modern descriptions now describe the multiple elements of the inferior longitudinal fasciculus (Fig. 3). One component comprises the core long fibre that is often thought of as the inferior longitudinal fasciculus, while the other component contains the groups of short fibres originally described by Tusa and Ungerleider (1985) that follow its course, connecting nearby gyri together (Catani *et al.*, 2003).

In the spirit of tracts being lost and found, most descriptions of the uncinate faciculus note a narrow U-shaped tract that connects the temporal pole to the orbitofrontal cortex. While this is one aspect of the uncinate that was described by the Dejerines, their uncinate fasciculus was a tract of greater complexity:

‘the innermost fibres of this fasciculus are as arced as the U-fibres that line the bottom of sulci; it is this pronounced curvature that earned it the name uncinate fasciculus. However, it is only the innermost fibres that possess such a pronounced curvature. The further away the fibres are from the anterior perforated substance (and this therefore concerns the more lateral fibres of the fasciculus), the less curved they become, so much so that the very end fibres are not only straight but are curved in the opposite direction.’

**Figure 4:**
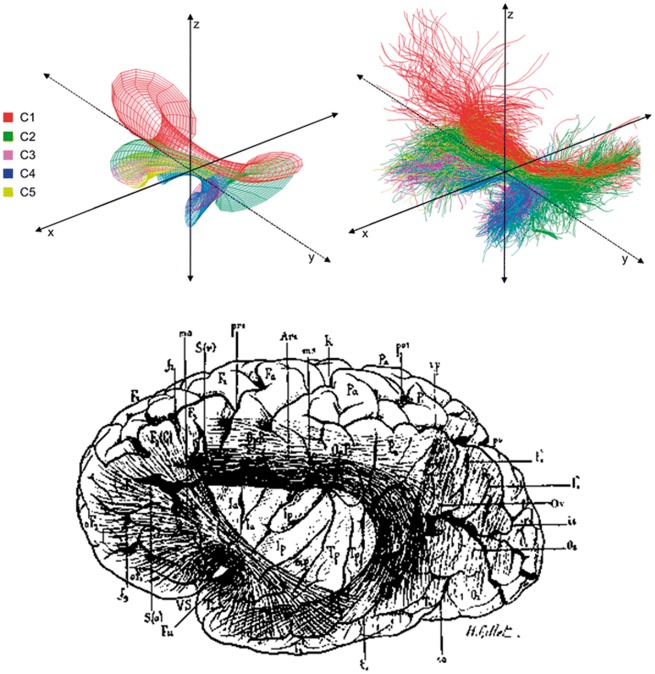
**The uncinate fasciculus.** *Top*: A reconstruction of the uncinate fasciculus by Hau *et al. *(2017). The different colours represent the graded retroflexing subcomponents of the tract. Reproduced with permission of Springer-Verlag Berlin-Heidelberg via Copyright Clearance Center. *Bottom*: An image from *Anatomie des centres nerveux* of the uncinate fasciculus (Fu) as described by the Dejerines. Note the similarities between the old and modern depictions.

Recently, Hau *et al.* (2017) revisited the uncinate fasciculus using both dissection techniques and tractography. They rediscovered the posterior aspect of the uncinate fasciculus and used cluster analysis to define five subcomponents of the tract. As can be seen in Fig. 4, the uncinate fasciculus described by Hau *et al.* (2017) mirrors the 19th century description by the Dejerines. While it is important to acknowledge that like all techniques, tractography is subject to a degree of potential error by way of false positive (and false negative) fibres, nevertheless, this rediscovery of a posterior (C1) component of the uncinate fasciculus reignites a conundrum in the literature as to whether the temporofrontal fibres that are held within this subcomponent constitute an independent tract, form part of the inferior-fronto-occipital fasciculus, are a subcomponent of the uncinate fasciculus, or—as indicated by the Dejerines—are a continuous, graded retroflexing of the uncinate fibres.

Indeed, more than one possibility may be correct: it may be that the posterior component of the uncinate may be both a curving of uncinate fibres as well as part of the inferior fronto-occipital fasciculus, as highlighted in the description provided by Gloor (1997):

‘[the occipito-frontal fasciculus] becomes closely associated with the most compact portion of the uncinate fasciculus, the two forming a double fan. The fasciulus occipito-frontalis inferior can thus be envisaged as representing a dorsal extension of the fronto-temporal associational system that forms the uncinate fasciculus.’

### The debate goes on

While some descriptions of the white matter anatomy found in *Anatomie des centres nerveux *will be familiar to the modern reader, many are steeped in controversy. The following section is devoted to the tracts that have stirred up debate in both the modern and the historical literature. Many of the tracts already described have been debated, discussed and redefined throughout history. However, some remain more controversial than others.

**Figure 5:**
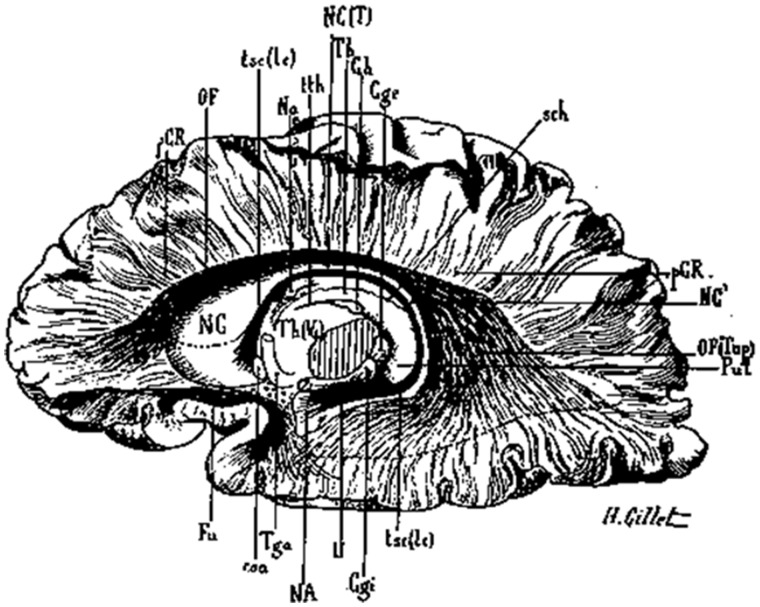
**An image from *Anatomie des centres nerveux* of the occipito-frontal fasciculus**.

The occipito-frontal fasciculus (Fig. 5) is a tract of particular contention (Schmahmann and Pandya, 2007). While first reportedly described by Forel and Onufrowicz in acallosal patients, the modern consensus is that this original description was of a heterotopic callosum rather than the fronto-occipital fasciculus (Forkel *et al.*, 2014). As such, the Dejerines are often credited as the first to describe an actual front-occipital fasciculus (Schmahman and Pandya, 2006). The modern literature, however, often refers to the Dejerines’ occipito-frontal fasciculus as the superior fronto-occipital fasciculus. This contrasts with a fasciculus that also connects the occipital lobe to the frontal lobe but which courses ventrally through the extreme capsule complex, and is often referred to as the inferior fronto-occipital fasciculus. Not all researchers, however, agree with this distinction, arguing that the occipito-frontal fasciculus of Dejerine is the only true fasciculus connecting these two lobes (Schmahmann and Pandya, 2007). In contrast, others maintain that the Dejerines’ occipito-frontal fasciculus is not an actual tract (Türe *et al.*, 1997), or posit that the superior fronto-occipital fasciculus is an ‘occipital extension’ of the superior longitudinal fasciculus (Forkel *et al.*, 2014). Furthermore, there has also been discussion as to whether the superior fronto-occipital fasciculus and the subcallosal bundle of Muratoff are the same tract, as implied by the Dejerines, or whether they are two separate bundles (Schmahman and Pandya, 2006). The occipito-frontal fasciculi, both superior and inferior, remain two of the modern neuroscience’s most often debated tracts.

### Nobody’s perfect

The brain is a complex structure and to date there has been no way to provide a truly objective, error-free description of its structure. Thus, anatomists of different eras may decide to give alternative names for the same structure, or may decide that the distinction is important. For example, the Dejerines used the terms arcuate fasciculus and superior longitudinal fasciculus interchangeably while modern neuroscientists make a distinction.

Furthermore, both classical dissection techniques as well as modern MRI techniques are error prone and user-dependent. Indeed, the challenges of white matter dissection may explain why the Dejerines’ description of the arcuate fasciculus (and other tracts), differs from modern conceptualizations that have brought together complementary evidence from a range of sources including tract tracer, electrophysiology, modern blunt dissections and tractography experiments. According to the Dejerines description:

‘The arcuate fasciculus appears to be composed of short association fibres that connect together two neighbouring gyri. Its deep layers, particularly those in contact with the external capsule, only contain a few longer fibres which, skipping over a gyrus, connect together two gyri a little further apart. But the arcuate fasciculus does not appear to contain fibres, of any length, that connect two distant lobes. In fact, we have seen several times, using a series of microscopic sections, that when the arcuate fasciculus or superior longitudinal fasciculus of Burdach is included in an old cortical lesion, there is hardly any degeneration of fibres beyond the immediate vicinity of the original source.’

**Figure 6:**
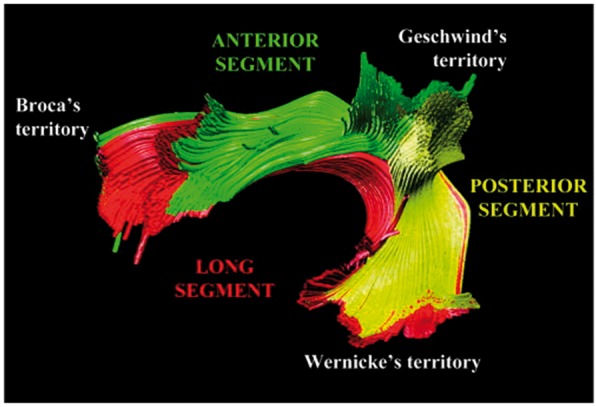
**A tractography reconstruction of the arcuate fasciculus by Catani *et al. *(2005) that shows three components of the arcuate fasciculus: two short components and one long segment. **The superior longitudinal fasciculus is now considered to be separate from the arcuate fasciculus and is itself also subdivided into three components. Reproduced with permission of John Wiley and Sons via Copyright Clearance Center. See Fig. 4 for a depiction of the arcuate (Arc) fasciculus as depicted by the Dejerines.

This description of the arcuate fasciculus as a collection of very short association fibres is in stark contrast to the modern view of the tract as a long-range connective pathway (Fig. 6). While individual components of the arcuate fasciculus are now recognized, including two ‘short’ components connecting posterior temporal to parietal and parietal to frontal regions, unlike the arcuate of the Dejerines, the tract is now considered to include an additional long component that courses through the entire fasciculus directly connecting frontal and temporal areas (Catani *et al.*, 2005).

## Reconnecting to the lasting legacy of the Dejerines

Aside from contending with archaic and occasionally cumbersome sentence structure, reading through the chapter on the long association fasciculi by Joseph and Augusta Dejerine feels almost modern, with some aspects being very familiar: they approached the subject by examining it through multimodal and convergent methodologies, and anatomy was often discussed in the context of the function that it underpins.

The MRI revolution in human neuroscience has opened the possibility of the widespread, repeated, *in vivo* study of human white matter, since we are now free from a reliance on the availability of post-mortem specimens. However, rarely does any advance come without its own challenges and limitations. Indeed, the easy availability of data can come at the cost of increasing abstraction and the validity of the tractography model is hard to verify. Furthermore, it is rare to find contemporary researchers who have investigated brain structures so thoroughly and systematically as did the Dejerines. Due to the relative dominance of the English language in the modern scientific literature and the relative lack of interest in historical sources, many researchers may not have access to the works of Dejerine (although we must acknowledge the efforts made by some authors to translate other important historical works) (Schmahmann and Pandya, 2006; Forkel *et al.*, 2014). Yet, in a field where descriptions and definitions of structures are of paramount importance, historical perspectives are vital.

While modern explorations of the white matter architecture have evolved from a traditional mapping of the large fibre tracts and their cortical origin/terminations to a more fine-grained delineation of their subdivisions, the focus has still been on the long-range association fibres. With current knowledge and methodological advances, we are now able to begin to explore both the short and long components, as well as their relationship to higher cognitive functions and the pattern of impairments that result when these are affected by different neurological diseases (Jung *et al.*, 2017).

Not only for an increased neuroscientific understanding, but also for a wider, especially clinical, application, the notions of the 19th century fasciculi remain important. These are the white matter bundles that are commonly affected by stroke and other forms of full depth injuries leading to various forms of disconnection syndromes (Catani and ffytche, 2005). They are also the white matter pathways that neurosurgeons see during surgery, and for which decisions must be made on whether to spare or to cut (Duffau, 2015).

In these situations, a comprehensive understanding of tract anatomy is essential. While the descriptions made by the Dejerines do not always conform to our modern understanding of tracts, their insights, taken in conjunction with modern evidence are still valuable. At the very least, the two volume *Anatomie des centres nerveux* is a beautiful and meticulous work which deserves appreciation. The Dejerines are one part of a long history of pioneers in neuroanatomy. In remembering them and their contribution, we can recognize that modern neuroscience is standing on the shoulders of giants.

## Supplementary Material

Supplementary Data

## References

[awx225-B1] Bajada CJ, Lambon Ralph MA, Cloutman LL Transport for language south of the Sylvian fissure: the routes and history of the main tracts and stations in the ventral language network. Cortex 2015; 69: 141–51. 26070011 10.1016/j.cortex.2015.05.011

[awx225-B2] Catani M, ffytche DH The rises and falls of disconnection syndromes. Brain 2005; 128: 2224–39. 16141282 10.1093/brain/awh622

[awx225-B3] Catani M, Jones DK, Donato R, Ffytche DH Occipito-temporal connections in the human brain. Brain 2003; 126: 2093–107. 12821517 10.1093/brain/awg203

[awx225-B4] Catani M, Jones DK, ffytche DH Perisylvian language networks of the human brain. Ann Neurol 2005; 57: 8–16. 15597383 10.1002/ana.20319

[awx225-B5] Dejerine JJ Contribution a l’étude anatomo-pathologique et clinique des différentes variétés de cécité verbale [Internet]. In: Masson G, editor. Comptes rendus des séances de la Société de Biologie et de ses filiales. Paris: la Société de Biologie; 1892 Available from: http://publikationen.ub.uni-frankfurt.de/frontdoor/index/index/docId/23183

[awx225-B6] Duffau H Stimulation mapping of white matter tracts to study brain functional connectivity. Nat Rev Neurol 2015; 11: 255–65. 25848923 10.1038/nrneurol.2015.51

[awx225-B7] Forkel SJ, Thiebaut de Schotten M, Kawadler JM, Dell’Acqua F, Danek A, Catani M The anatomy of fronto-occipital connections from early blunt dissections to contemporary tractography. Cortex 2014; 56: 73–84. 23137651 10.1016/j.cortex.2012.09.005

[awx225-B8] Gloor P The temporal lobe and limbic system. New York: Oxford University Press; 1997.

[awx225-B9] Hau J, Sarubbo S, Houde JC, Corsini F, Girard G, Deledalle C, Revisiting the human uncinate fasciculus, its subcomponents and asymmetries with stem-based tractography and microdissection validation. Brain Struct Funct 2017; 222: 1645–62. 27581617 10.1007/s00429-016-1298-6

[awx225-B10] Jones DK, Christiansen KF, Chapman RJ, Aggleton JP Distinct subdivisions of the cingulum bundle revealed by diffusion MRI fibre tracking: implications for neuropsychological investigations. Neuropsychologia 2013; 51: 67–78. 23178227 10.1016/j.neuropsychologia.2012.11.018 PMC3611599

[awx225-B11] Jung J, Cloutman LL, Binney RJ, Lambon Ralph MA The structural connectivity of higher order association cortices reflects human functional brain networks. Cortex 2017, in press. [doi: 10.1016/j.cortex.2016.08.011] Available from: http://ac.els-cdn.com/S0010945216302325/1-s2.0-S0010945216302325-main.pdf?_tid=c887cd62-87f0-11e7-b7be-00000aacb362&acdnat=1503485568_0ee31f9f42d3f0ab7a10568a90af22c0 10.1016/j.cortex.2016.08.011 PMC5726605 27692846

[awx225-B12] Schmahmann JD, Pandya D Fiber pathways of the brain. New York: Oxford University Press, USA; 2006.

[awx225-B13] Schmahmann JD, Pandya DN The complex history of the fronto-occipital fasciculus. J Hist Neurosci 2007; 16: 362–77. 17966054 10.1080/09647040600620468

[awx225-B14] Schurch B, Dollfus P The “Dejerines”: an historical review and homage to two pioneers in the field of neurology and their contribution to the understanding of spinal cord pathology. Spinal Cord 1998; 36: 78–86. 9494995 10.1038/sj.sc.3100561

[awx225-B15] Türe U, Yaşargil MG, Pait TG Is there a superior occipitofrontal fasciculus? A microsurgical anatomic study. Neurosurgery 1997; 40: 1226–32. 9179896 10.1097/00006123-199706000-00022

[awx225-B16] Tusa RJ, Ungerleider LG The inferior longitudinal fasciculus: a reexamination in humans and monkeys. Ann Neurol 1985; 18: 583–91. 4073852 10.1002/ana.410180512

