# Corrigendum

**DOI:** 10.1093/brain/awx266

**Published:** 2017-10-11

Claude J. Bajada, Briony Banks, Matthew A. Lambon Ralph, Lauren L. Cloutman. Reconnecting with Joseph and Augusta Dejerine: 100 years on. Brain 2017; 140: 2752-2759. 10.1093/brain/awx225.

The title of the above manuscript should have read: Reconnecting with Joseph and Augusta Dejerine: 100 years on.

This has now been corrected online, and the authors apologize for the error.

