# Supplementary material for: Reconnecting with Joseph and Augusta Dejerine: 100 years on
Source: Brain. 2017 Sep 15;140(10):2752–9. doi: 10.1093/brain/awx225 (PMC5841156; doi:10.1093/brain/awx225)
Supplement: Supplementary Data [file awx225_supp.pdf]

Supplementary Materials

Translation of Anatomy of the Central Nervous System; Section on the Long Association Fasciculi (pp. 749 – 780)

Authors: Joseph Jules Dejerine & Augusta Dejerine-Klumpke
First Published: 1895
Translators: Claude J Bajada and Briony Banks

Translators' note

The following is a translation of a section on the long association tracts from Anatomy of the Central Nervous System by Joseph Jules Dejerine and Augusta Dejerine-Klumpke from the original French to English. This translation was done as a collaboration between a neuroscientist and medical doctor with an interest in history (CJB) and a neuroscientist with a French language degree (BB). The translation was primarily carried out by CJB using various dictionaries and translators including Google translate and Linguee.com, with secondary translation and editing support by BB. All medical and terminology, particularly antiquated ones were checked and verified by checking different sources. However, where there was doubt or ambiguity a footnote was entered. The preliminary translation was then scrutinised for linguistic accuracy and for anatomical and historical consistency (by CJB, BB, LC and MALR).

To the extent that is possible, the Dejerines' style was retained. Where words were replaced for readability then a footnote was inserted. Formatting such as italicization was kept matching that in the original text. Figure numbers and legend references were also retained from the original in order to allow the reader to refer to the original text that can be found freely online: https://archive.org/details/anatomiedescentr01dj

There are three types of footnotes that can be found in this text. The first type is additional translator annotations to the main text that the translators felt would be of benefit to the readers. The second type of footnote highlights the original French term that was used in the Dejerines' text. This was done when, although a particular English word was deemed to be most suited in the context, it does not necessarily capture the full meaning of the original French. Finally, some footnotes are used when the translators updated terminology to fit modern standards with the footnote stating the precise term that the Dejerines used. All three types of footnotes are explicitly differentiated.

It is hoped that this translation, along with the accompanying Commentary, will honour the great work done by two 19th century masters of anatomy on the 100th anniversary of Joseph Dejerine's death.

The Long Association Fasciculi

There are five long association fasciculi: the cingulum; the arcuate, or superior longitudinal, fasciculus; the inferior longitudinal fasciculus; the uncinate fasciculus and the occipito-frontal fasciculus of Forel and Onufrowicz.

The *cingulum* is the association fasciculus of the rhinencephalon. It connects the first limbic gyrus[1] to the second[2]. Furthermore, it connects the limbic lobe to the medial and possibly lateral surface (Beevor[3]) of the hemisphere.

The other four fasciculi belong to the cerebral cortex and serve to connect the different lobes. The *superior longitudinal fasciculus* (or *arcuate fasciculus*) connects the frontal lobe to the parietal and temporal lobes, passing over the Sylvian operculum. The *uncinate fasciculus* passes through the limen insulae and connects the temporal lobe to the frontal lobe. The *inferior longitudinal fasciculus* connects the temporal lobe to the occipital lobe. The *occipito-frontal fasciculus* of Forel and Onufrowicz forms the tapetum and provides a connection between the occipito-temporal lobe and the frontal lobe.

The Cingulum (Burdach) (Cing), *the longitudinal fasciculus of the gyrus fornicalus[4], peripheral fornix[5]* (Arnold) (See fig. 373, 374 and Macro and Microscopic Coronal Cross Sections, chap. II and III). The *cingulum* is the long association fasciculus of the rhinencephalon. It is an arced fasciculus, in the sagittal direction, that is located on the medial surface of each hemisphere. To a large extent, it forms the white matter of the first and second limbic gyri (L1 and L2 [H][6]). Arnold erroneously believed it to be a single entity in the hippocampal gyrus (as though it formed a single system with the fornix[7]), and split at the level of the corpus callosum. He called the fornix the *internal fornix*, and the cingulum the *peripheral fornix*. He believed that these two formations were connected through fibres crossing the corpus callosum.

The *cingulum* is easy to expose in a brain fixed in alcohol or dichromate[8] by removing the cortex and the *U-fibres* of the first limbic gyrus (L1). It appears as an arced fasciculus which passes over the rostrum, genu, body and splenium of the corpus callosum, narrows at the isthmus of the limbic lobe (L[i]) and then spreads out and swells at the hippocampal gyrus (L[H_2]), passing inferiorly and anteriorly towards the anterior end of the uncus[9] of the temporal lobe (U). Dissections show that the cingulum is not formed of fibres that extend to the full length of the fasciculus, but of relatively short fibres. These short fibres are curved at both ends to penetrate the white matter of the surrounding gyri

---

1 Now known as the cingulate gyrus
2 Now known as the hippocampal gyrus
3 The names referred to throughout the chapter are the researchers whose work was under discussion at the time and were cited by the Dejerines
4 The gyrus fornicalus (from Latin fornicatus - arched) is formed by the concatenation of the cingulate gyrus and the hippocampal gyrus to form a complete arch.
5 Original: Fornix periphericus
6 The letters appearing throughout the chapter refer to the labels found in the original diagrams.
7 This is referred to by the Dejerines as the cerebral trigone.
8 Potassium or ammonium
9 Original: la circonvolution du crochet

and, for part of their trajectory, constitute the cingulum of Burdach. The fasciculus, in effect, receives and emits fibres to and from the superior frontal gyrus[10] (mF_1), the paracentral lobule (Parc), the precuneus (PrC), the cuneus (C), the lingual lobe (Lg), the fusiform lobe (Fus) and the temporal pole. This continual receipt and emission of fibres explains why the width of the cingulum varies so little as it runs along the upper surface of the corpus callosum; indeed, the cingulum only narrows at the level of the pre-calcarine isthmus.

In a coronal cross section of a hemisphere fixed in dichromate (fig. 238-261 and 280-288), the cingulum looks like a dark, clearly defined, pear-shaped[11] fasciculus that occupies the inferior half of the first limbic gyrus (L). Its base rests on top of the corpus callosum (Ce) at the point where it penetrates the white matter of the centrum semiovale. The vertex of the cingulum extends to the peak of the first limbic gyrus (L). The inner edge of the fasciculus is concave and cradles the taenia tecta[12] (tec) and the cortex of the first limbic gyrus, from which it is separated by the neighbouring short association fibres[13]. The outer, or convex, edge of the fasciculus closely abuts the fibres of the centrum ovale (CO) and is penetrated by callosal fibres that extend towards the superior aspect of the hemisphere and the medial gyri.

According to Meynert, Schwalbe, Obersteiner, etc., the most infero-medial fibres of the cingulum were not covered by cortex, but formed, on the superior surface of the corpus callosum, the *nerves of Lancisi*, the *taenia tecta* and, at the level of the hippocampal gyrus ($H[L_2]$), the *reticular substance of Arnold* (Lms). Now, we know that these formations (the *nerves of Lancisi,* the *taenia tecta* and the *reticular substance*) are completely distinct from the cingulum; in fact, as we have seen earlier, they form the region's cortical tangential fibres (p. 699-701).

Posterior to the splenium of the corpus callosum (Ce[Spl]), the cingulum is penetrated by fibres of the *forceps major* that project to the cuneus (C) and the lingual lobule (Lg). At the pre-calcarine isthmus (L[i]), the narrowed section of the cingulum is in contact with the most anterior fibres of the stratum calcarinum[14] (strK) that line the common branch of the calcarine and parieto-occipital fissures (K + po). Finally, in the hippocampal gyrus (L_2 [H]), cingulum fibres heavily stained with haematoxylin run in a sagittal direction and cap the medial end of the diverticulum of the subiculum (Cing [p]) (Figure 351, p.706).

The origin and termination of the cingulate fibres are highly controversial. For Foville, both ends of the cingulum terminate in the anterior perforated substance. For Broca, they connect the medial olfactory root to the lateral olfactory root, and the author compares the cingulate system to a racket of which the olfactory tract represents the handle. For Meynert, Huguenin, etc., the antero-inferior end of the cingulum connects to the amygdala.

[10] Referred to by the Dejerines as the 'first frontal gyrus'
[11] Original: piriformis
[12] Also called the indusiuim griseum – the layer of grey matter that covers the dorsal aspect of the corpus callosum
[13] U-fibres
[14] The U-shaped fibres that connect the upper and lower banks of the calcarine sulcus

Finally, for Beevor, the cingulum consists of three independent fasciculi: the *anterior, horizontal* and *posterior fasciculi* (fig. 373, 374). The *anterior fasciculus* (Cing [a]), located below the genu and rostrum of the corpus callosum, connects the anterior perforated substance, and particularly the medial olfactory root (Roli), with the anterior end of the frontal lobe; none of its fibres curve around the genu of the corpus callosum to enter the horizontal fasciculus.

The *horizontal fasciculus* (Cing[h]) runs along the superior aspect of the corpus callosum. Its fibres originate from the frontal extremity of the hemisphere (both medial and lateral surfaces), and connect the first limbic gyrus (L_1) to the medial gyri and probably also to the lateral gyri.

Finally, the *posterior fasciculus* (Cing[p]) is situated in the hippocampal gyrus (H [L_2]), which it connects to the lingual gyrus (Lg), the fusiform lobule (Fus) and the gyri of the temporal pole. No fibres run superiorly towards Ammon's horn or the amygdala, nor do they run towards the anterior perforated substance (Beevor).

The *function* of the cingulum is rather obscure. Horsley severed the fasciculus anterior to the precuneus in marmoset (Hapale jactans) and found no form of paralysis or anaesthesia. The animal was sacrificed two months after the operation and, on examination of the brain, Beevor observed degeneration of fibres in the posterior section of the horizontal fasciculus[15]. Not every fibre in this section had degenerated, a fact which supports the currently accepted opinion that fibres in the cingulum do not extend along its full length; rather, it contains fibres that only belong to the fasciculus for a single part of their trajectory.

The Uncinate Fasciculus. (Fu) (fig. 375, 376, 377 and 381). Discovered by Reil, the *uncinate* or *hooked fasciculus,* the *unciform fasciculus of Burdach,* is the shortest of the long association fasciculi of the cerebral cortex. It is easy to expose by dissecting a brain fixed in alcohol or dichromate. It connects the temporal pole to the orbital surface of the frontal lobe and occupies the limen insulae, where it extends in the transverse direction, from the anterior perforated substance to the extreme capsule.

Since the orbital surface of the frontal lobe is very close to the temporal pole, the innermost fibres of this fasciculus are as arced as the U-fibres that line the bottom of sulci; it is this pronounced curvature that earned it the name *uncinate fasciculus*. However, it is only the innermost fibres that possess such a pronounced curvature. The further away the fibres are from the anterior perforated substance (and this therefore concerns the more lateral fibres of the fasciculus), the less curved they become, so much so that the very end fibres are not only straight but are curved in the opposite direction (fig. 376).

The uncinate fasciculus does not stain well using the haematoxylin methods of Pal and Weigert, and it is very dense at the level of the limen insulae. In coronal sections (fig. 281-283), it has the shape of

[15] Original: faisceau horizontal

an irregular fasciculus, bound by the extreme capsule (Cex) and the anterior perforated substance (Spa). Located outside the anterior commissure (coa), it intersects with the inferior longitudinal fasciculus (Fli) and divides the horizontal section of the claustrum[16] (AM') near to the posterior marginal sulcus of the insula (mp).

At the *frontal end* (fig. 375), the most inferior, medial and superficial fibres of the uncinate fasciculus run anteriorly and medially, passing between the anterior perforated substance and the inferior surface of the putamen. Near the olfactory sulcus, they intersect with fibres from the genu of the corpus callosum, and terminate at the medial section of the superior frontal gyrus.

Subsequent fibres either run into the gyrus rectus (oF_1 [Gr]), or to the orbital section of the superior and inferior frontal gyri (oF_1, oF_3). Finally, the most lateral fibres curve superiorly and laterally, intersect with fibres from the genu of the corpus callosum, and terminate with these fibres at the peak of the orbital and lateral sections of the inferior frontal gyrus (oF_3, F_3[c]).

At the *temporal end*, the fibres of the uncinate fasciculus divide the grey matter that connects the claustrum (AM) to the amygdala (NA) and to the temporal cortex, at the level of the anterior perforated substance (fig. 283). They intersect with the inferior longitudinal fasciculus (in microscopic sections coloured using Weigert and Pal's methods, fibres of the uncinate fasciculus stain less darkly than those of the inferior longitudinal fasciculus thus making them distinct), then radiate into the uncus (U), the temporal pole and the anterior sections of the superior and middle temporal gyri[17] (T_1, T_2); at this level, they intersect with the superior longitudinal fasciculus (Arc).

The uncinate fasciculus is often affected by lesions in the insula and in the external capsule, and its degenerated fibres can be traced along the temporal pole and the orbital part of the frontal lobe (see next chapter, Vol. II).

The Superior Longitudinal or Arcuate Fasciculus (Arc) *(fasciculus arcuatus de Burdach)* (fig. 376, 377). The *arcuate fasciculus* is found on the lateral surface of the hemisphere, while the cingulum is on the medial surface. It is located at the base of the gyri that form the Sylvian operculum, exterior to the fibres at the base of the corona radiata and level with the trunk of the corpus callosum, (see fig. 382 and *Macroscopic Coronal Sections*, fig. 247-250). This fasciculus follows a wide arc that runs inferiorly and anteriorly, similar to the cingulum and uncinate fasciculus. In dissections of brains fixed in dichromate or alcohol, the arcuate fasciculus only appears to be dense in the parietal region of the operculum; parallel to the superior edge of the putamen, these fibres, which are the most inferior and superficial, reach the superior marginal sulcus of the insula, split apart the superior edge of the claustrum, cover the *exterior* fibres of the base of the corona radiata, and continue to form the superior section of the external capsule. At the level of the posterior end of the Sylvian fissure (S[p]), the fasciculus forms an anteriorly concave curve which cradles the posterior edge of the putamen; its

[16] Original: l'avant-mur
[17] Referred to by the Dejerines as the first and second temporal gyri

fibres then fan out, intertwine with the fibres of the corona radiata and of the splenium of the corpus callosum, and for some time run alongside the base of the parieto-occipito-temporal gyri. The most superficial fibres run anteriorly, cover the exterior of the uncinate fasciculus (Fu) and radiate into the peak of the anterior section of the superior temporal gyrus (T_1). The next layer of fibres terminates in the posterior section of the superior temporal gyrus (T_1) and in the middle temporal gyrus (T_2), where the fibres intertwine with fibres of the corona radiata and the splenium of the corpus callosum. Finally, the deepest fibres radiate into the peaks of the supramarginal gyrus (Gsm), the angular gyrus (Pc)[18] and the gyri of the lateral surface of the occipital lobe (O_1, O_2, O_3). Moving away from the posterior marginal sulcus of the insula (mp), these fibres become more and more difficult to follow as they become interwoven with the callosal and projection fibres.

On coronal sections of brain fixed in alkaline dichromate (see fig. 382 and Macroscopic Sections, fig. 242-255), the cross-sectional area of the arcuate fasciculus is triangular. Its infero-medial angle corresponds to the external capsule and threads itself between the latter and the claustrum; its infero-lateral angle corresponds to the base of the Sylvian opercular gyri, while its superior angle is lost in the layer of surrounding fibres. It is poorly defined at the lower exterior,[19] where it merges imperceptibly with the white matter of the Sylvian opercular gyri, but it is very well defined at the interior, where it touches the base of the corona radiata[20]: indeed, the direction of its fibres is perpendicular to those of the corona radiata. At the level of the supramarginal gyrus, the arcuate fasciculus curves inferiorly and anteriorly to reach the temporal lobe. It merges backwards with the layer of vertical fibres forming the convexity of the occipital and parieto-temporal regions, a layer formed by the *vertical occipital fasciculus* (Ov, fig. 377) of Wernicke.

On microscopic sections treated using Weigert or Pal's methods, the arcuate fasciculus stains faintly with haematoxylin and is difficult to distinguish from the white matter of the base of the Sylvian opercular gyri.

The termination of the front end of the arcuate fasciculus is highly debated. According to Meynert it ends in the rolandic operculum (OpR) and the operculum of the inferior frontal gyrus (OpF_3), and constitutes an association fasciculus that connects the lateral surface of the temporo-occipital regions to the convexity of the frontal lobe.

For Schnopfhagen, on the other hand, the front end of the arcuate or superior longitudinal fasciculus does not terminate at the level of the inferior frontal gyus (F_3); at this level, it intertwines with the fibres of the corona radiata, then runs anteriorly and medially towards the anterior section of the corpus callosum, crosses the midline at the genu of the corpus callosum, and terminates in the frontal lobe of the contralateral hemisphere. Thus, for this author, the arcuate fasciculus belongs to the system of the corpus callosum, and connects the temporo-occipital lobe of one hemisphere with the frontal lobe of the contralateral hemisphere. This is a question that can only be resolved by developmental

[18] Original: le pli courbe
[19] Original: … en dehors et en bas
[20] Original: où il est appliqué sur le pied de la couronne rayonnante

anatomy and secondary degeneration; nevertheless, cases of agenesis of the corpus callosum do not appear to support Schnopfhagen's hypothesis.

Moreover, the arcuate fasciculus appears to be composed of short association fibres that connect together two neighbouring gyri. Its deep layers, particularly those in contact with the external capsule, only contain a few longer fibres which, skipping over a gyrus, connect together two gyri a little further apart. But the arcuate fasciculus does not appear to contain fibres, of any length, that connect two distant lobes. In fact, we have seen several times, using a series of microscopic sections, that when the *arcuate fasciculus* or *superior longitudinal fasciculus* of Burdach is included in an old cortical lesion, there is hardly any degeneration of fibres beyond the immediate vicinity of the original source.

The Occipito-Frontal Fasciculus[21] (*Forel and Onufrowicz*) (OF). Forel and Onufrowicz demonstrated that, in complete agenesis of the corpus callosum, arrested development involves the corpus callosum (rostrum, genu, body and splenium), the forceps and the commissural system of the fornix (Lyra Davidis) (fig. 378, 379, 380) – a fact that was confirmed by Kaufmann and Hochhaus. On the other hand, the *tapetum* – that is, the layer of fibres that lines the outer wall of the sphenoid and occipital horns [of the lateral ventricles] – is normally developed, and continues anteriorly along a sagittal fasciculus, located medial to the corona radiata and external to the body of the fornix to which it is closely related (fig.379). These facts therefore reveal that the tapetum does not belong to the corpus callosum (as was believed until the work of Forel and Onufrowicz), but to an intrahemispheric association fasciculus which these authors named the *occipito-frontal fasciculus*. In a normal hemisphere, to which fasciculus does this occipito-frontal fasciculus (that formed, at the level of the ventricular atrium, the *tapetum* of previous authors), correspond?

Forel and Onufrowicz identified it as the *superior longitudinal* or *arcuate fasciculus of Burdach*, and their view was accepted by Kaufmann and Hochhaus. We cannot agree with this view; in fact, we have just observed that the superior longitudinal fasciculus of Burdach is located *exterior [lateral]* to the corona radiata, and that its most inferior fibres cover the exterior of the external capsule. In contrast, the occipito-frontal fasciculus is located *medial to* the corona radiata and forms the roof of the lateral ventricle (fig. 379 and 380).

Sachs hypothesizes that, in cases of arrested development of the corpus callosum, it is not a question of agenesis, but of a kind of heterotopia of the corpus callosum. The callosal fibres are developed, but instead of running transversally from lateral to medial and linking the two hemipheres, they run within the same hemisphere from the posterior to the anterior, becoming a sagittal fasciculus. Yet one can hardly understand how, or under what influence, such a transformation would come about. Congenital cerebral agenesis concerns arrested development not only of the fibres and their terminals, but also of the originating cells of a system of neurons. The situation is comparable to that observed in

[21] Note that this is not the inferior-fronto-occipital fasciculus that dominates the modern literature but a dorsal pathway that is sometimes referred to as the superior fronto-occipital fasiculus.

Guddon's experimental approach, where lesions[22] in *newborn* animals result in the complete disappearance of the fibres and their originating cells, without leaving any residue (Forel).

In our opinion, the *occipito-frontal fasciculus* of Forel and Onufrowicz is identifiable as a sagittal fasciculus that, in a normal hemisphere, runs along the external angle of the lateral ventricle. This fasciculus is located in the interior of the corona radiata, above the caudate nucleus, below and exterior to the corpus callosum; it is separated from the ventricle by subependymal grey matter. This fasciculus, which we named the *occipito-frontal fasciculus* (OF) in our series of sections, corresponds to the fasciculus alternately described by Meynert as the *corona radiata of the caudate nucleus,* and by Wernicke as the *fasciculus of the corpus callosum that leads to the internal capsule*.

According to Meynert, the *corona radiata of the caudate nucleus* is formed of many fibres that originate in the caudate nucleus, emerging along its supero-lateral edge and radiating into the gyri of the superior edge of the hemisphere. Yet, Wernicke showed that these fibres do not form a single connection with the caudate nucleus; on examination of microscopic serial sections, we see, in fact, that none of these fibres terminates in the caudate nucleus.

According to Wernicke, these fibres constitute a *fasciculus of callosal fibres that lead to the internal capsule* (Balkenbündel zur inneren Kapsel). They originate from the anterior wall of the frontal horn, that is, from the genu of the corpus callosum and from the white matter of the frontal lobe. The fibres converge to form a compact fasciculus measuring a centimetre and a half thick. It runs along the superolateral edge of the caudate nucleus and its fibres penetrate the internal capsule, between the caudate nucleus and the superior edge of the putamen, at the level of the middle segment of the optic radiation. This fasciculus is not therefore part of the anterior segment of the internal capsule (Wernicke).

On examination of microscopic serial sections, whether coronal, axial or sagittal, we were convinced time and again that not one fibre from this fasciculus connects with the caudate nucleus. Since no anatomopathological or experimental evidence supports Gratiolet and Foville's former opinion (which was revived by Wernicke), on the existence of callosal fibres leading to the internal capsule, we identified this fasciculus as the *occipito-frontal fasciculus* described by Forel and Onufrowicz in cases of agenesis of the corpus callosum.

It is possible that, in cases of agenesis of the corpus callosum, the cingulum joins together with the occipito-frontal fasciculus, but the cingulum does not, on its own, constitute this fasciculus. The cingulum runs along the first limbic gyrus and is reflected back in the hippocampal gyrus, but none of its fibres enter the external wall of the sphenoidal and occipital horns [of the lateral ventricles].

Hence, we describe a long association fasciculus that we have named the *occipito-frontal fasciculus*, that runs in a sagittal direction and is situated between the cingulum and the arcuate (or superior longitudinal) fasciculus of Burdach. It is separated from the cingulum by the entire thickness of the

[22] Described as extirpations

corpus callosum, and from the superior longitudinal fasciculus by the base of the corona radiata (fig. 381, 382). Like other long association fasciculi that we have just studied, the occipito-frontal fasciculus follows a wide arc that runs anteriorly and inferiorly. The whole of its surface area is covered by the ependyma and subependymal grey matter, to which it emits numerous fibres. This fasciculus runs along the external angle of the lateral ventricle and is located superior to the caudate nucleus, within the corona radiata, inferior to the hooked path that the callosal fibres follow around the external angle of the lateral ventricle.

In coronal sections (fig. 382), its cross-sectional area is pear-shaped[23] and is approximately half a centimetre thick. Its base rests on the corona radiata, while its apex, directed superiorly and medially, threads itself between the callosal fibres and the ventricular ependyma. Clearly defined at the level of the head and body of the caudate nucleus, the fasciculus is partially split at the level of the tail of the caudate nucleus, by fibres from the corona radiata and corpus callosum. On reaching the level of the ventricular atrium, this fasciculus curves inferiorly and anteriorly, and its fibres fan out across the infero-external wall of the sphenoidal horn, forming the *tapetum* described by previous authors.

Anteriorly, the *occipito-frontal fasciculus* originates from the entire frontal cortex – the lateral surface, the frontal pole and the orbital surface. Along the way, it receives a large number of fibres from the superior edge of the hemisphere and from the gyri of the lateral surface, which reach the fasciculus by passing between the fibres of the corpus callosum and the corona radiata. This is easily observed on microscopic coronal (fig. 382) or axial sections (fig. 301 to 304 and 295, 296), that are prepared using Weigert and Pal’s method. The fibres of the occipito-frontal fasciculus form bundles that are clearly distinct from one another, and stain less intensely with haematoxylin than do the fibres of the corpus callosum or the corona radiata. The main fasciculus emits a large number of fine, faintly-stained fibres, which project anteriorly and medially (horizontal sections fig, 295) or supero-medially (coronal sections fig. 382), and occupy all the clear space, including between the fibres of the corona radiata and the corpus callosum surrounding the lateral ventricle. Other fibres (OF’, fig. 391, p. 790) cross the subependymal grey matter, intersect with fibres of the corpus callosum and the corona radiata anterior to the frontal horn [of the lateral ventricle], and radiate into the anterior extremity of the frontal lobe. On coronal cross-sections, we can also see fibres emanating from the base of the occipito-frontal fasciculus which run infero-laterally, crossing the base of the corona radiata and entering the body of the *external capsule*.

In the spheno-occipital lobe[24], after having formed the tapetum, the fibres of the occipito-frontal fasciculus radiate into the gyri of the lateral surface and the infero-lateral edge of the lobe. It is likely that they intersect with the fibres of the *forceps major of the corpus callosum* at the level of the ventricular atrium, and in particular at the level of the occipital horn. By examining a series of coronal sections, we can very clearly see fibres emanating from the *forceps major* and following the lateral

---

[23] Original: pyriform

[24] A satisfactory translation for the spheno-occipital lobe is not apparent. It is suspected that the Dejerines are referring to the basal occipitotemporal junction at the point where the spheno-occipital synchondrosis occurs (base of skull).

wall of the occipital horn [of the lateral ventricle]. In lesions of the occipital lobe, we can further see the degenerated fibres of the tapetum partly penetrating the forceps major (fig. 394). These observations seem to indicate that the corpus callosum makes up a certain part of the lateral wall of the occipital horn; this is all the more likely since Cajal showed that the callosal fibres are often only the medial bifurcate branch of the long association fibres.

As we shall see later, lesions of the occipital lobe affect not only the forceps of the corpus callosum, but also the tapetum and the occipito-frontal fasciculus. Furthermore, the latter fasciculus can partially degenerate as a result of lesions in the temporal lobe and in the convexity of the hemisphere, and its degenerated fibres can be traced in the subependymal grey matter. We report examples of degeneration that is cortical in origin in subsequent chapters (see. Vol. II).

These facts, which we have observed in humans are, furthermore, in keeping with what we can learn from experimental pathology. In experimental research conducted on dogs by Muratoff, ablation of the motor area[25], or destruction of the frontal or occipital gyri, resulted in the partial degeneration of the occipito-frontal fasciculus of Forel and Onufrowicz (the *subcallosal fasciculus* of Muratoff). *But this fasciculus, and particularly the tapetum, remains intact after severing the corpus callosum, when the cortex is not accidentally damaged during the operation.* The independence of the tapetum and callosal system, demonstrated by Forel and Onufrowicz using teratology, is therefore confirmed by experimental pathology. Experimentation thus provides us with an outcome that the method of secondary degeneration cannot, since the callosal, association and projection fibres degenerate together following cortical lesions.

The *occipito-frontal fasciculus* therefore constitutes a long association fasciculus which connects the temporo-occipital lobe to the frontal lobe, to the convexity of the hemisphere, and to the insula (by fibres leading into the external capsule). Like all long association fasciculi, it is formed of fibres of unequal length that only belong to the *occipito-frontal fasciculus* for part of their trajectory. This fasciculus can only be distinguished from the other long association fasciculi by its deep and subependymal location; it is, in effect, located *medial to* the projection system, while the other long association fasciculi occupy an eccentric position relative to this system.

The Inferior Longitudinal Fasciculus (Burdach) (Fli). *Sagittal layers of the occipital lobe* (Wernicke). *External sagittal stratum* (Sachs). *Sensory fasciculus* (Charcot, Ballet) (fig. 373, 374, 383, etc.).

The *inferior longitudinal fasciculus* of Burdach runs in an antero-posterior direction, and is located at the level of the infero-lateral edge of the spheno-occipital[26] lobe extending from the occipital to the temporal pole. It forms the *external layer of sagittal fibres* in the occipito-temporal lobe, and is

[25] The original text refers to this structure as the 'sphère motrice' which literally translates as the motor sphere. We have been deliberately vague in this translation as the original term leaves some ambiguity. One potential for the structure being referred to is the basal ganglia.
[26] This is most likely describing the section of the brain where the occipital lobe meets the medial portion of the temporal lobe that underlies the sphenoid bone.

extremely easy to expose in a brain fixed in alcohol or in alkaline dichromate, because the direction of its fibres are perpendicular to those that cover it (fig. 383 and 389).

As Burdach has shown, this fasciculus forms, as a whole, a sort of trough or sharply angled groove which is open superiorly and medially, and which receives projection fibres from the occipito-temporal lobe into its concavity. It affords a lateral wall, an inferior wall, and a very thick salient angle which corresponds to the infero-lateral edge of the hemisphere, particularly the base of the *inferior temporal gyrus*[27] (T_3) and the *inferior occipital gyrus*[28] (O_3) (fig. 383, see also figs. 252 to 262). The lateral wall runs alongside the *superior* and *middle temporal gyri*[29] (T_1, T_2); the inferior wall borders the diverticulum of the subiculum (ds) inferiorly, lines the base of the *fusiform lobule* (Fus) and the *lingual lobule* (Lg), and merges medially with the *posterior fasciculus of the cingulum* (Cing [p]), situated in the *hippocampal gyrus* (H[L_2]).

However, the inferior longitudinal fasciculus only has this particular form in the temporal lobe. In the occipital lobe, the inferior wall of the trough is directed superiorly and medially and, at the level of the calcar avis,[30] it lines the stratum calcarinum (fig. 384). At the base of the cuneus, the two edges of the trough are so close to each other that they join together, transforming this fasciculus into a type of hollow cone, of which the tapered peak is situated approximately two and a half centimetres from the occipital pole. This formation means that, on coronal sections, the *inferior longitudinal fasciculus* takes the shape of a somewhat irregular and angular ring in the occipital lobe (fig. 384 and 290), while in the temporal lobe it takes the shape of a sharply angled trough (fig. 383). The *inferior longitudinal fasciculus* is therefore a clearly defined fasciculus, although some authors have erroneously used the term to describe all of the commissural, projection and association fibres that line the floor of the sphenoid and occipital horns [of the lateral ventricles], and that lie between the ventricular ependyma and the cortex of the collateral sulcus.

The thickness of the *inferior longitudinal fasciculus* varies according to region. Its posterior section, where it forms a complete ring around the tapered end of the occipital horn, is thin. It becomes considerably thicker along the lateral wall and the infero-lateral edge of the ventricular atrium (where it condenses into a dense fasciculus of very tightly knit fibres), while at the level of the calcar avis it shrinks to a very thin layer of fibres that can only be detected under a microscope.

Its concave surface rests against the layer of *temporo-occipital lobe projection fibres*, also known as the *internal sagittal layer* and the *thalamic radiations*, which separates the inferior longitudinal fasciculus from the tapetum (Tap) and the ventricular cavity (Vl).

Its convex surface is uneven and is covered by association fibres specific to the occipital lobe (see p. 780). At the base of each gyrus that borders the convex surface, a sort of crest projects out, formed from intersecting commissural, association and projection fibres at the base of the gyri. These crests

[27] Referred to by the Dejerines as the third temporal gyrus
[28] Referred to by the Dejerines as the third occipital gyrus
[29] Referred to by the Dejerines as the first and second temporal gyri
[30] Original: l'ergot de Morand

are in the sagittal direction like the gyri of the occipito-temporal lobe; they are not very pronounced at the level of the angular gyrus and the second parietal gyrus, but are particularly accentuated at the level of the three temporal gyri (T_1, T_2 and T_3), the third occipital gyrus (O_3), and the fusiform (Fus) and lingual lobules (Lg) (fig. 384, 385, see also figs. 223 to 228, 251 to 266, 273 to 276). Indeed, the inferior longitudinal fasciculus emits a very large number of fibres to these gyri.

The crest of the lingual gyrus has been described by Burdach as the *medio-basal fasciculus* (inneres Grundbündel) (fig. 373 and 374, Fbi). It corresponds to the infero-medial section of the inferior longitudinal fasciculus, reinforces, anteriorly, the *posterior fasciculus of the cingulum* (located within the hippocampal gyrus, H[L_2]) at the level of the retro-limbic fold,[31] and disappears with the retro-limbic fold.

The crests that cross the convex surface of the inferior longitudinal fasciculus are very clear in coronal sections, whether one studies macroscopic brain slices fixed with dichromate, or microscopic sections, treated with carmine or using Weigert or Pal's methods. They are also easy to observe in axial sections where the temporal gyri are cut obliquely (fig. 385 and 293).

In the temporal lobe, the inferior longitudinal fasciculus is poorly defined superiorly. Indeed, it merges with projection fibres of the temporal and parietal lobes at the level of the retro-lenticular section of the internal capsule, that it covers exteriorly (fig. 383, 385 and p. 532, fig. 287). This configuration means that the fasciculus is only visible as a clearly distinct layer in regions located posterior and inferior to the curved section of the tail of the caudate nucleus (see Series I, II, III, p. 536, fig. 283 to 288, 292, 293 and p. 593, figs. 305 to 314).

Anterior to the retro-lenticular section of the internal capsule (Cirl), the inferior longitudinal fasciculus surrounds the posterior extremity and the inferior edge of the putamen (NL_3) (fig. 385). It then bypasses the sphenoidal horn [of the lateral ventricle] (Vsph) and reaches the external part of the amygdala (NA); here, along with the uncinate fasciculus, it breaks up the grey matter that connects the claustrum to the amygdala and to the neighbouring cortex (fig. 386).

In sections treated using Weigert or Pal's methods, the inferior longitudinal fasciculus is intensely stained by haematoxylin. It is clearly distinguishable from the thalamic radiations (RTh) and from the white matter fibres of the temporo-occipital lobe due to the large calibre and the intense staining of its fibres, which combine into dense fascicles that are separated from each other – firstly, by fine, lightly stained fibres most likely belonging to Gratiolet's thalamic radiations, and secondly, by darkly stained fibres that cross the inferior longitudinal fasciculus, perpendicular to the direction of its fibres, and penetrate the tapetum.

Like all long association fasciculi, the inferior longitudinal fasciculus comprises a complex system of fibres of unequal lengths. However, secondary degeneration, resulting from localised cortical lesions of the occipital lobe, has shown us that this fasciculus contains a large number of long fibres whose degeneration can be seen in the white matter of the temporal lobe.

[31] Original: le pli rétro-limbique

The fibres of the inferior longitudinal fasciculus originate in the cortex of the occiptal pole and lobe. They cross the white matter of the lobe radially, along with the projection and commissural fibres, and group together early on, posterior to the occipital horn, to form a thin annular fasciculus which is easy to distinguish in sections treated using Weigert's method (p. 560 fig. 290, and p. 768 fig. 384). Along the way, this fasciculus receives a large number of fibres from the cuneus (C), the lingual gyrus (Lg), the fusiform lobule (Fus) and from the three lateral occipital gyri (O_1,O_2,O_3).

These fibres do not all run in the same direction. Fibres that originate from the inferior and lateral half of the occipital lobe run from posterior to anterior along the infero-lateral edge of the sphenoid and occipital horns [of the lateral ventricles]. However, fibres arising from the supero-lateral part of the occipital lobe run very obliquely, inferiorly and anteriorly, along the external wall of the ventricular atrium, while fibres originating in more superior regions follow an even more oblique course.

Similarly, fibres that originate from the cuneal cortex run obliquely, inferiorly and anteriorly, along the medial wall of the occipital horn [of the lateral ventricle] exterior to the *stratum calcarinum,* then pass under the floor of the occipital horn. All of these fibres thus get closer and closer to the dense fasciculus of sagittal fibres, which runs along the infero-lateral edge of the ventricular atrium, and which takes the shape of a trough in the temporal lobe.

On reaching the level of the temporal lobe, the *inferior longitudinal fasciculus* emits many fibres to the lobe's gyri. The inferior layers of fibres enter the hippocampal gyrus (H[L_2]), the fusiform gyrus (Fus) and the inferior temporal gyrus (T_3); many radiate into the middle temporal gyrus (T_2), while an even greater number radiate into the superior temporal gyrus and reach the temporal pole, where they intersect with the fibres of the arcuate, or superior longitudinal fasciculus of Burdach, and with those of the uncinate fasciculus.

A small number of fibres penetrate the external capsule, with which they merge to form the most inferior layers. These fibres, which scarcely go beyond the posterior third of the external capsule (fig. 303 to 326), intersect with fibres from the anterior commissure and the uncinate fasciculus, but are distinguishable from them due to their larger calibre and intense staining.

Finally, other fibres radiate into the cortex of the uncus (U), in the temporal cortex that borders the anterior perforated substance (Epa) posteriorly, and in the adjacent cortex of the superior temporal gyrus (T_1) (fig. 386) (see also the series of *axial sections* III and IV, p. 587 to 657, fig. 303 to 326).

It is easy to observe where the inferior longitudinal fasciculus ends anteriorly, and where it entwines with the uncinate fasciculus, in horizontal serial sections (p. 734, fig. 375).

In microscopic axial (fig. 383) or coronal sections (fig. 387), the inferior longitudinal fasciculus has a very particular appearance near the superior temporal gyrus: it is crossed by a large number of undulating fasciculi, intensely stained with haematoxylin, which bend anteriorly and medially, cross the *thalamic radiations*, penetrate the *retro-lenticular section* of the *internal capsule*, then radiate into

the *pulvinar*, the *lateral and medial geniculate body*, and the *lateral and medial thalamic nuclei*[32]. The most anterior of these undulating fascicles reach the posterior segment of the internal capsule in the region of the *subthalamic nucleus*, and descend, with the fibres of the posterior segment, into the *base* of the *cerebral peduncle*[33], with which they merge to form its lateral fifth.

Do these fibres, that cross the thalamic radiations and the retro-lenticular section of the internal capsule, belong to the inferior longitudinal fasciculus, part of which penetrates the corona radiata? Or do they instead represent projection fibres from the temporal lobe which only cross the inferior longitudinal fasciculus to get to their destination? It is important to address this question because it has implications for the function of the inferior longitudinal fasciculus. The latter hypothesis implies that this fasciculus is exclusively an association fasciculus, while the former would suggest that it contains both association and projection fibres. Several authors have likened the fasciculus to the projection fibres of the occipital lobe, describing it thus using the names *optic radiations* (Gratiolet), *lamina of the optic fasciculus* (Meynert), *sagittal substance of the occipital lobe* (Wernicke), and *sensory fasciculus*[34] (Charcot, Ballet, Brissaud); according to these authors, the inferior longitudinal fasciculus is only formed of projection fibres.

For Sachs, the layer of fibres that he refers to as the *sagittal stratum externum* is mainly formed of association fibres, but also contains a small number of projection fibres.

It is indisputable that the projection and association fibres of the inferior longitudinal fasciculus cannot be distinguished from one another using normal anatomy alone: the intertwining of the fibres is too great, their intersections too many. The problem can only be solved using secondary degeneration, comparative anatomy, or by other methods. In our opinion, the inferior section of the inferior longitudinal fasciculus is exclusively an association fasciculus. With regard to its superior section, which is closely linked to the retro-lenticular segment of the internal capsule, it certainly contains projection fibres that run to subcortical centres (thalamus, lateral geniculate body, globus pallidus, etc.).

Earlier, we stressed the fact that, throughout its occipital section, the inferior longitudinal fasciculus is crossed by fibres that run from the occipital cortex to the tapetum and the thalamic radiations. We believe that the same is true in the temporal section of this fasciculus. We believe that most of the fibres emitted from the sagittal layer of the occipito-temporal lobe, that radiate into the pulvinar, the medial and lateral geniculate nuclei, the lateral thalamic nucleus, the sub-optic region, and the posterior segment of the internal capsule, etc. do not belong exclusively to the inferior longitudinal fasciculus; in fact, they only cross the fasciculus and belong either to the *corona radiata of the occipital lobe*, or to the *corona radiata of the temporal lobe*.

The fibres of the *temporal lobe's corona radiata* (that is, the projection fibres of the temporal lobe) pass under the inferior surface of the putamen (NL_3), between this surface and the curved end of the

[32] The thalamus described as the 'couche optique' in the Encyclopedie Larousse
[33] This refers to the crus cerebri
[34] Original: le faisceau sensitif

tail of the caudate nucleus (NC) (fig. 387), and within the whole of the area that lies between the retrolenticular segment of the internal capsule (Cirl) and the amygdala (NA) (Cip' fig. 214, p. 370). The corona radiata of the temporal lobe projects to the thalamus,[35] the medial geniculate body, the pulvinar, the globus pallidus, the base of the cerebral peduncle, etc.; thus, it must cross the superior section of the inferior longitudinal fasciculus before reaching its destination. The projection fibres of the temporal lobe that run down to the base of the cerebral peduncle provide proof of this proposition.

By studying the topography of secondary degeneration in five cases of old lesions in the temporal cortex, on microscopic serial sections, one of us [the authors] has in fact shown that the fibres which make up the lateral fifth (or thereabouts) of the base of the cerebral peduncle (that is, the fibres of the "so-called *Türck's* fasciculus[36]"), originate not from the occipital lobe, as stated by Türck, Meynert, Wernicke, Charcot, Ballet, Brissaud, etc., but from the temporal lobe. In fact, *Türck's fasciculus* degenerates as a result of localised lesions in the temporal cortex, particularly the middle part of the middle and superior temporal gyri; in these cases, one sees its degenerated fibres cross the superior part of the inferior longitudinal fasciculus, pass under the third segment of the lenticular nucleus and reach, in the superior subthalamic region, the posterior segment of the internal capsule, immediately anterior to its retrolenticular segment.

Studying secondary degeneration as a result of lesions in the occipital cortex confirms the *temporal* origin of Türck's fasciculus. One of us [the authors] carried out research on five cases of lesions in the occipital cortex (affecting either the occipital convexity up to the angular gyrus inclusively, or the cuneus, the lingual lobule, or the fusiform lobule) using microscopic serial sections. This research showed that the base of the cerebral peduncle and the posterior segment of the internal capsule remain intact following lesions in the occipital lobe and that, in these cases, only the retrolenticular segment of the capsule is affected. Vialet, in his research on the *Cerebral Centres of Vision*, achieved the same results. The description of degeneration in the five cases of hemianopia studied in this important work effectively shows that, as long as the lesion is limited to the occipital lobe, secondary degeneration is always confined to the retrolenticular segment of the internal capsule; it affects the subcortical optic centres, the pulvinar, the lateral geniculate nucleus and the brachium of the superior colliculus, but does not affect at all the base of the cerebral peduncle.

Conversely, once the cortical damage is no longer confined to the occipital lobe, but impinges on the parietal and temporal lobes, the topography of the capsular degeneration changes: the retrolenticular segment is no longer the only damaged section, as the degenerated area extends to the posterior segment of the internal capsule and continues on to the base of the cerebral peduncle.

From the research that we have just described it therefore follows that, contrary to the opinion of Meynert which was also accepted by Huguenin, Charcot, Ballet and Brissaud, the inferior longitudinal fasciculus (as well as the optic radiations that it neighbours), does not send any fibres into the inferior part of the cerebral peduncle.

[35] Original: la couche optique
[36] Now known as the anterior corticospinal tract

Isolated from, or associated with, the optic radiations, the inferior longitudinal fasciculus has no role in the transmission of somatosensory information, contrary to what was accepted by Meynert, Charcot and Ballet. For the latter two authors, the inferior longitudinal fasciculus, along with Gratiolet's optic radiations, represented the fasciculus that conducted general sensation, the *sensory* fasciculus, which fanned out into the occipital lobe and constituted the lateral fasciculus of the cerebral peduncle. For Brissaud, who adopts the ideas of the previous authors, the inferior longitudinal fasciculus represents a lateral sensory fasciculus, and the optic radiation represents a medial sensory fasciculus, both terminating in the lateral fasciculus of the cerebral peduncle or in Türck's fasciculus.

Today, this idea can no longer be accepted because, on the one hand, as we have just indicated, Türck's fasciculus does not originate in the occipital lobe, and on the other hand, lesions in the occipital lobe, cortical or sub-cortical, do not affect overall sensation – this has been proven through numerous clinical observations followed by autopsies. (See Vol. II, *Reil's Ribbon* and *Cerebral Localisations*[37].)

Türck's fasciculus is not the only projection fasciculus of the temporal lobe that intersects with the inferior longitudinal fasciculus. Other fibres from the temporal lobe's corona radiata, such as the *radiations of the medial geniculate nucleus*, the *temporal fibres* destined for the pulvinar, and the lateral thalamic nucleus[38], etc., also cross the inferior longitudinal fasciculus and contribute to the special appearance of its anterior end. The radiations of the medial geniculate nucleus originate in the superior temporal gyrus, as Monakow, Mahaim, etc., have shown. We have seen some cases of secondary degeneration, arising from lesions in the temporal cortex (see Vol. II), that cross the inferior longitudinal fasciculus and the retrolenticular segment of the internal capsule, and reach the section of the medial geniculate body that is deeply embedded in the thalamus[39].

The same is true of temporal fibres that are destined for the pulvinar, and that we will refer to as *temporo-thalamic fibres*. In a series of axial cross sections, which were fortunately obliquely oriented in a direction from superior to inferior, posterior to anterior and medial to lateral (these cross sections were even more oblique than our series IV, p. 633, fig. 316 to 328), we can investigate in the same way the length of temporal lobe fibres that connect the anterior extremity of the lobe to the pulvinar. These fibres come together in the lateral and superior part of the *amygdala*, are interlaced with the inferior longitudinal fasciculus along their whole length, pass beneath the lenticular nucleus and run, at an oblique angle, posteriorly, medially and superiorly towards the pulvinar.

Meynert's infero-medial thalamic[40] *peduncle,* whose fibres pass through Reichert's innominate substance and run obliquely *anteriorly* and medially, is not identified with the *temporo-thalamic fibres,* whose path is very easy to follow in cases of degeneration of the inferior longitudinal fasciculus (see Vol. II, Horizontal Sections, in relation to figs. 396 to 398, p. 798 [Vol. I]).

---

[37] Original: *Ruban de Reil* et *Localisations cérébrales*
[38] Original: "… au noyau externe de la couche optique"
[39] Original: la couche optique
[40] Original: "le pédoncule inféro-interne de la couche optique de Meynert"

On examination of microscopic serial sections of monkey's brain (see fig. 388), one finds the same highly characteristic differences in the staining of the sagittal stratum[41] that one finds in humans. At first glance, there seems to be only one sagittal stratum (RTh, fig. 388), corresponding to the fusion of the thalamic radiations (*the internal sagittal stratum*) and the inferior longitudinal fasciculus (*the external sagittal stratum*), which is formed of tightly packed fibres arranged in dense fasciculi, that are *strongly stained* by hematoxylin. These fibres originate in the occipital lobe (the occipital pole, medial and lateral surfaces); they run from posterior to anterior, receive a large number of fibres from the calcar avis[42], completely surround the occipital horn, and merge together along the lateral wall of the ventricle. They then curve round medially, at the level of the tail of the caudate nucleus, to radiate into the thalamus and in particular the pulvinar and the lateral geniculate body, which is highly developed in this animal (fig. 388).

However, on closer inspection, we note that between this layer of fibres that are strongly stained by haematoxylin, and the *vertical occipital fasciculus* of Wernicke (see fig. 388, Ov), there is a very thin layer of fine fibres that are weakly stained by haematoxylin (fig3 388, Fli), arranged in loose fascicles, and which must, in our opinion, be identified with the inferior longitudinal fasciculus in humans.

On microscopic serial sections, the fasciculus originates posterior to the occipital gyri, particularly in the occipital pole and its medial and lateral surfaces. From there it runs anteriorly and surrounds the thick layer of the thalamic radiations, forming a thin but complete layer. On sections of inferior temporal regions, its fibres radiate anteriorly into the temporal gyri, the amygdala, and the cortex that borders the anterior perforated substance posteriorly. On higher sections where the basal ganglia and the thalamus[43] are visible, the majority of fibres of the inferior longitudinal fasciculus radiate anteriorly into the temporal gyri, particularly the superior temporal gyrus; a small number cap the posterior extremity of the putamen and run into the external capsule.

Apart from its small size and the fact that it stains faintly with haematoxylin, the inferior longitudinal fasciculus is similar to that in humans. The weak staining is not surprising, given the lack of development of white matter in monkeys, in particular the intra-hemispheric association fibres.

In summary, secondary degeneration and comparative anatomy thus show that the *inferior longitudinal fasciculus* is primarily an association fasciculus that connects the occipital lobe, particularly the visual area, to the temporal lobe. This fasciculus degenerates following lesions in the occipital lobe and is concerned with a variety of word blindness that one of us [the authors] has described as *pure word blindness*. But, as with all association fasciculi, it is formed of fibres of unequal length that only belong to this fibre bundle as part of their complete trajectory. It therefore follows that degeneration never affects the entire thickness of this fasciculus, and as we move away from the source of the lesion, healthy fibres join the degenerated fibres.

---

[41] Original: la couche sagittale
[42] Original: l'ergot de Morand
[43] Collectively referred to as 'les corps opto-striés' in the original text

When studying secondary degeneration in the inferior longitudinal fasciculus, following lesions that do not involve the occipital cortex, but the white matter underlying it and, in particular, lesions located in the sagittal stratum of the temporo-occipital lobe, one sees that this fasciculus, as with the optic radiations, degenerates in both directions: we have seen this very clearly in two cases. It can therefore be asked if the inferior longitudinal fasciculus contains (apart from the fibres that come from the occipital cortex and constitute virtually the rest of the fasciculus) a number of fibres which, arriving from the temporal cortex, branch out into the occipital cortex. The method of secondary degeneration is incapable of resolving this issue, as we know from Gudden's experimental method (see p. 12 and 178) and from pathological anatomy, that encephalic and medullary fasciculi become damaged in both directions. The method of secondary degeneration, which is perfectly reliable for determining the path of a particular fasciculus, is in fact much less reliable when it comes to recognising its cellular origin.
